# Lanai: A small, fast growing tomato variety is an excellent model system for studying geminiviruses

**DOI:** 10.1016/j.jviromet.2018.03.002

**Published:** 2018-06

**Authors:** C.A. Rajabu, G.G. Kennedy, J. Ndunguru, E.M. Ateka, F. Tairo, L. Hanley-Bowdoin, J.T Ascencio-Ibáñez

**Affiliations:** a Department of Plant and Microbial Biology, North Carolina State University, Raleigh NC, 27695, USA; b Department of Horticulture, Jomo Kenyatta University of Agriculture and Technology, Nairobi, Kenya; c Department of Entomology and Plant Pathology, North Carolina State University, Raleigh NC, 27695, USA; d Mikocheni Agricultural Research Institute, Dar es Salaam, Tanzania; e Department of Molecular and Structural Biochemistry, North Carolina State University, Polk Hall 132, Box 7622, NCSU Campus, Raleigh NC, 27695, USA

**Keywords:** Florida lanai, Tomato, Geminiviruses, Symptoms, qPCR, Ploidy, Seed transmission

## Abstract

Geminiviruses are devastating single-stranded DNA viruses that infect a wide variety of crops in tropical and subtropical areas of the world. Tomato, which is a host for more than 100 geminiviruses, is one of the most affected crops. Developing plant models to study geminivirus-host interaction is important for the design of virus management strategies. In this study, “Florida Lanai” tomato was broadly characterized using three begomoviruses (*Tomato yellow leaf curl virus,* TYLCV; *Tomato mottle virus,* ToMoV; *Tomato golden mosaic virus,* TGMV) and a curtovirus (*Beet curly top virus,* BCTV). Infection rates of 100% were achieved by agroinoculation of TYLCV, ToMoV or BCTV. Mechanical inoculation of ToMoV or TGMV using a microsprayer as well as whitefly transmission of TYLCV or ToMoV also resulted in 100% infection frequencies. Symptoms appeared as early as four days post inoculation when agroinoculation or bombardment was used. Symptoms were distinct for each virus and a range of features, including plant height, flower number, fruit number, fruit weight and ploidy, was characterized. Due to its small size, rapid growth, ease of characterization and maintenance, and distinct responses to different geminiviruses, “Florida Lanai” is an excellent choice for comparing geminivirus infection in a common host.

## Highlights

• Florida Lanai is a tomato variety suitable for virus-host interaction studies.
• Florida-Lanai was infected by geminiviruses delivered by different methods.
• Florida-Lanai shows distinct measurable symptoms for different geminiviruses.
• Florida-Lanai has a small size, rapid growth and is easy to maintain.
• Florida-Lanai is an excellent choice for comparing geminivirus infections.

## 1 Introduction

Geminiviruses belong to a large, diverse family of plant infecting viruses (*Geminiviridae*) that are transmitted by insects and cause economically significant diseases worldwide (Zhang et al., 2001; Rojas et al., 2005; Hanley-Bowdoin et al., 2013). Geminiviruses are among the most economically important pathogens in a variety of crops including vegetables, fruits, root crops, cereals, spices and legumes (Morales and Anderson, 2001; Mansoor et al., 2003; Seal et al., 2006). The genomes of geminiviruses consist of either one (monopartite) or two (bipartite) circular, single-stranded DNA molecules, with the components of bipartite viruses known as DNA-A and DNA-B (Zhang et al., 2001; Brown et al., 2012; Hanley-Bowdoin et al., 2013). Geminiviruses are classified in nine genera according to their genome, host and insect vector (Zerbini et al., 2017).

Management of plant viruses is of vital importance to reduce the damage (Sastry and Zitter, 2014), especially in areas where food security is at risk due to high viral diversity and the emergence of more virulent strains (Damsteegt, 1999; Mansoor et al., 2003; Sastry and Zitter, 2014). In 2009, Rodrigues et al. (Rodrigues et al., 2009) concluded that disease management strategies need extensive knowledge of the virus infection, transmission, spread and their effects on host plants to select the best control measures. Studying viruses can be simplified if a tractable host system is available. The suitability of a host for studying the infection process is determined by its ability to become infected and to allow the virus to replicate and induce typical symptoms (Scholthof et al., 1996).

Geminiviruses, have been studied using model plant systems such as *Arabidopsis thaliana* (Muangsan et al., 2004; Ascencio-Ibáñez et al., 2008; Hanley-Bowdoin et al., 2013; Raja et al., 2014), *Nicotiana benthamiana* (Goodin et al., 2008), *Solanum nigrum* (Urbino et al., 2008), and *Datura stramonium* (Chen et al., 2013). These model plants have many advantages including small size, short life cycles, high seed germination rates and ease of genetic analysis (Meissner et al., 1997; Meinke et al., 1998; Matsukura et al., 2008). For example, *Arabidopsis* has one of the smallest genomes, making it useful for genetic manipulation (Bevan and Walsh, 2006). Model plants are also usually inexpensive to study and readily accessible. However, information obtained using model plants may not always accurately reflect viral interactions or processes that occur in a non-model crop or reservoir plants in nature and disease can be the result of specific interactions between a virus and a host (Dawson and Hilf, 1992; Pallas and Garcia, 2011).

Of the 322 begomoviruses recognized by the International Committee on Taxonomy of Viruses, more than a third infect tomato and probably many others can infect solanaceous plants, underscoring the importance of having a suitable tomato variety for virus testing. Tomato (*Solanum lycopersicum* L., *Solanaceae*) is an herbaceous plant with hundreds of varieties that differ in size and generation time. Tomato has long been the preferred system for studying plant-pathogen interactions involving plants from the *Solanaceae* family (Arie et al., 2007; Meissner et al. 1997; Emmanuel and Levy, 2002). Tomato is susceptible to a wide range of viral diseases, many of which are associated with significant agronomic losses (Hanssen et al., 2010; Inoue-Nagata et al., 2016). As an example, tomato yellow leaf curl disease is caused by begomoviruses and has spread worldwide to become one of the most important viral diseases of tomato (Lefeuvre et al., 2010).

There is considerable physiological and genetic variation among tomato varieties that affects their suitability for laboratory studies. Among tomato varieties, Micro-Tom (TGRC accession # LA3911, UC Davis, Department of Plant Sciences, USA), a dwarf tomato cultivar derived from crossing cv. Florida Basket and Ohio 4013-3 (Scott and Harbaugh, 1989), is widely used in laboratory studies due to its small size (15–20 cm in height), rapid life cycle (70–90 days), and because it can be readily and efficiently transformed (Emmanuel and Levy, 2002; Meissner et al., 1997, Martí et al., 2006; Carvalho et al., 2011; Okabe et al., 2011; Sun et al., 2006). Studies require less time to complete because of its rapid life cycle that can accommodate up to four generations per year. Even though Micro-Tom has been widely adopted, its potential for molecular studies is limited because of its mutant genetic background, which results in brassinosteroid deficiency and deep green rugose leaves induced by the presence of the *dwarf (d)* and *miniature (mnt)* recessive genes (Bishop et al., 1996; Pnueli et al., 1998; Martí et al., 2006). The brassinosteroid pathway has been implicated in viral disease and symptom development, and alterations in the pathway may interfere with virus-plant interaction studies in Micro-Tom (Campos et al., 2010). Moreover, the gibberellin response is altered in Micro-Tom (Martí et al., 2006) and further interferes with data interpretation. In addition, Micro-Tom has a mutation in the *self-pruning (sp)* gene, which controls the regularity of the vegetative-reproductive switch along the compound shoot of tomato. This mutation is responsible for its determinate phenotype (Pnueli et al., 1998). Thus, it is important to look for new model systems that are either alternative or complementary to those currently used.

*S. lycopersicum* ‘Florida Lanai’ is also a small tomato variety that was developed for home gardens (Augustine et al., 1981). It has regular leaves and determinate growth, reaching a height of 60–90 cm. Flowers are open pollinated and produce a medium sized fruit (under 450 g) maturing approximately 60 days after transplanting or 90 days from seeding. Seed germination rate ranges from 82% to 96%. Even though ‘Florida Lanai’ plants are small and have a short generation time, they do not carry the recessive genes that place the use of Micro-Tom in doubt. ‘Florida Lanai’ has been used previously to characterize a new begomovirus species (Tomato yellow margin leaf curl virus) using biolistics to inoculate infectious clones (Nava et al., 2013). It has also been used to study geminivirus-insect interactions (McKenzie, 2002), although there has been no systematic characterization of its suitability as a model system for geminiviruses. In this study, we used three inoculation methods to examine ‘Florida Lanai’ as a model system for studying diverse geminiviruses that naturally infect tomato.

## 2 Materials and methods

### 2.1 Plant growth conditions and inoculation protocols

Florida Lanai seeds were kindly supplied by J. Scott (University of Florida, USA). ‘Florida Lanai’ plants were grown in sterile soil from seeds in a walk-in growth chamber at 25 °C, 80% humidity and a 16:8 light/dark (LD) cycle. After one week, the seedlings were transplanted into pots and propagated for two more weeks before inoculation. Virus inoculation was done by either *Agrobacterium*-mediated inoculation, low-pressure particle acceleration DNA delivery using a microdrop sprayer (Venganza, Inc.) or whitefly transmission from infected to healthy plant. The infectious clones corresponding to *Beet curly top virus* (BCTV), *Tomato yellow leaf curl virus* (TYLCV), *Tomato mottle virus* (ToMoV DNA-A and DNA-B), *Tomato golden mosaic virus* (TGMV DNA-A and DNA-B), *Cabbage leaf curl virus* (CaLCuV DNA-A and DNA-B), are described in Table 1. *E. coli* cultures for TYLCV, ToMoV, TGMV and CaLCuV DNA A and DNA B were prepared in LB broth containing 0.1 μg/ml carbenicillin, subsequently grown overnight at 37 °C with vigorous shaking. Similarly their corresponding *Agrobacterium* clones were prepared in LB broth containing 0.075 μg/ml Spectinomycin grown at 30 °C. For BCTV, *E. coli* and *Agrobacterium* clones were prepared in 0.05 μg/ml kanamycin LB broth cultured overnight at their respective temperatures. All experiments were repeated three times.

**Table 1:** Infectious viral clones used to inoculate ‘Florida Lanai’ plants by agroinoculation or biolistics.

Virus^a^	Plasmid used for biolistics	Plasmid used for agroinoculation	References and comments
BCTV	BCTV in pMON521	BCTV in pMON521	Beet curly top virus (BCTV; strain Logan), a pMON525-based plasmid containing a BCTV DNA containing a partial tandem copy (provided by D. M. Bisaro of Ohio State University, Stenger et al., 1992).
TYLCV	pTYLCV2	pNSB1736	Partial tandem copy of Tomato yellow leaf curl virus (TYLCV; Dominican Republic isolate) cloned into pMON721 (Settlage et al., 2005; Reyes et al., 2013), from Acc. number AF024715.
ToMoV DNA A	pNSB1906	pNSB1906	Partial tandem copy of Tomato mottle virus (ToMoV) DNA-A cloned into pMON721 (Abouzid et al., 1992, Reyes et al., 2013)
ToMoV DNA B	pNSB1877	pNSB1877	Partial tandem copy of Tomato mottle virus (ToMoV) DNA-B cloned into pMON721 (Abouzid et al., 1992, Reyes et al., 2013)
TGMV DNA A	pMON1565	pMON337	Partial tandem copy of Tomato golden mosaic virus (TGMV) DNA-A (Fontes et al., 1994; Orozco and Hanley-Bowdoin, 1996; Elmer et al., 1988).
TGMV DNA B	pTG1.4B	pMON393	Partial tandem copy of Tomato golden mosaic virus (TGMV) DNA-B cloned in pTG1.4B (Fontes et al., 1994; Orozco and Hanley-Bowdoin, 1996).
CaLCuV DNA A	pCpCLCV A.003	pNSB1090	Cabbage leaf curl virus (CaLCuV) with a partial tandem copy (Turnage et al., 2002; Egelkrout et al., 2002).
CaLCuV DNA B	pCpCLCV B.003	pNSB1091	Cabbage leaf curl virus (CaLCuV) with a partial tandem copy (Turnage et al., 2002; Egelkrout et al., 2002).

a All clones have been designed to contain two viral origins of replication which allow the vector to release a functional viral monomer circularized by Rep and identical to wild-type viral DNA.

#### 2.1.1 Agrobacterium-mediated inoculation

*Agrobacterium* cultures containing infectious clones in binary vectors were grown in LB broth with their corresponding antibiotics at 30 °C overnight. The bacterial cultures were diluted 10-fold with LB media and used to inoculate ten plants for each treatment. For bipartite viruses, equal amounts of cultures corresponding to DNA-A and DNA-B genomes were mixed prior to inoculation. An *Agrobacterium* strain carrying an empty T-DNA vector was used for mock inoculation. Plants were then returned to the growth chamber. Agroinoculation procedures were described previously by Reyes et al, (2013).

#### 2.1.2 Biolistics

Plasmid DNA (5 μg) carrying infectious clones was coated onto 1 μm gold (Au) particle suspensions as described in Cabrera-Ponce et al. (1997). The final pellet was resuspended in 65 μL of absolute ethanol and used to spray 6 plants (10 μL/plant) at 40 psi. For the bipartite geminiviruses, 5 μg of each viral DNA component were mixed prior to coating the gold particles. The sprayer was positioned 2.5 cm from the plant apex. Empty plasmid DNA was used for the mock controls.

#### 2.1.3 Whitefly transmission

Experiments were carried out in whitefly proof cages using *Bemisia tabaci* MEAM1 adults from a colony maintained on ‘Florida Lanai’ tomato at 27 °C and a 16:8 LD cycle in an environmental chamber. Approximately 100 adult whiteflies between 2 and 10 days post-eclosion were allowed to acquire virus by caging for 72 hr with a symptomatic ‘Florida Lanai’ plant infected with either TYLCV or ToMoV. The whiteflies were transferred to new cages containing healthy ‘Florida Lanai’ plants and allowed to feed continuously. The mock treatment was done by feeding the whiteflies on healthy plants. The plants were inspected for symptoms at 28 days post inoculation (dpi) and leaf samples collected for PCR analysis.

#### 2.1.4 Seed transmission

Seeds were harvested from plants showing typical symptoms of TYLCV, ToMoV, BCTV or TGMV. Harvested seeds were washed, dried and sown in new pots. Samples were taken for DNA isolation from one leaflet of the fourth compound leaf (counted from the top of the plant) at 3 and 6 weeks after planting from 6 plants per treatment. Equal amounts of DNA from 6 plants were pooled for each treatment. For BCTV-infected plants, which do not produce fruit if infected early, healthy plants were inoculated with BCTV after initial fruit-setting. Seeds were harvested and analyzed as described above. All pooled samples were analysed by conventional PCR using virus-specific primers (Table 2).

**Table 2:** List of primers used for PCR amplification of viruses in this study.

Primer name	Sequence (5′ → 3′)	Virus species	Expected size (nt)
BCTV15-for^a^	CGTTACTGTGACGAAGCATTTG	BCTV	283
BCTV15-rev^a^	CTCCTTCCCTCCATATCCAGTA	BCTV	
TYLCV15-for^b^	CCTCTGGCTGTGTTCTGTTATC	TYLCV	257
TYLCV15-rev^b^	GCAATCTTCGTCACCCTCTAC	TYLCV	
ToMoV pNSB1^c^	GTCCAATACTCTCTCGTCCAATC	ToMoV	239
ToMoV pNSB2^c^	CAGCGGCCTTGTTAATTCTTG	ToMoV	
Sal-Nco^d^	CGACAAAGACGGAGATACTCT	TGMV	397
AL1 RT^d^	GCCTAGTGAACGAGCCCACA	TGMV	
CaLCuV1990-F^e^	ACATACATCAGAGTCGCAAGAG	CaLCuV	223
CaLCuV1990-R^d^	ACTGCCCGGATTCACAATAA	CaLCuV	

a Primer designed using GenBank accession nos. NC_001412, M24597, AY134867, EU586260 and JN817383.

b Primer designed using GenBank accession nos. AM409201, EU085423, AB192965, KC852149 and KJ879950.

c Primer designed using GenBank accession nos. EF028241, L14460, EU709520 and AY965900.

d Primer designed using GenBank accession nos. K02029, JF694490 and JF694488.

e Primer designed using GeneBank accession nos. U65529 and DQ178612.

### 2.2 Disease, growth and yield monitoring

Plants were inspected weekly from 1 dpi to record disease symptoms and plant height. Disease symptoms were recorded by photography using a digital camera (Panasonic Lumix DMC-FZ28). Plant height (cm) was measured from the base to the tip of the main shoot for each plant (Olaniyi et al., 2010). The measurements were recorded as height increase by subtracting initial height of a plant at the time of inoculation from the height measured at the time of data recording. Data were also recorded on yield parameters (number of flowers, number of fruit and fresh fruit weight). The number of flowers was recorded 60 days after planting. The number of fruit and the fresh fruit weight was recorded at harvest (95 days from planting).

### 2.3 DNA extraction and virus detection

Samples were collected from the fourth compound leaf from the top of individual plants, and consisted of a single base leaflet. Independent samples were placed in 2-mL cryovials at 14, 17, 21 and 31 dpi from 10 plants for each treatment and frozen immediately in liquid nitrogen. DNA was extracted using the CTAB DNA extraction method (Doyle and Doyle, 1987). DNA concentrations and quality were assessed using a Nanodrop (Thermo Scientific™). For plants infected with ToMoV, which showed a recovery phenotype, DNA was prepared from the first, second and third compound leaves from the apex.

A convergent primer pair that amplifies a short DNA fragment (≤300 bp) was designed for each virus (Table 2). Primers were first tested in conventional PCR to establish optimum annealing temperature and amplification efficiency before being used in quantitative real-time PCR (qPCR). Viral DNA was quantified using a qPCR standard curve generated by amplification of known amounts of plasmid DNA containing viral sequences (Table 1) that was 10-fold serially diluted from 10^−1^ to 10^−4^ range. The concentration of the template DNA in the reaction mix was converted from ng/μL to copy number/μL using the following formula: (C × 10^−9^/MW) × NA where C = template concentration ng/μL, MW = template molecular weight in Daltons and NA = Avogadro's constant, 6.022 × 10^23^. MW was obtained by multiplying the number of base pairs of a plasmid by the average molecular mass of one base pair (660 g/mol). A base 10 logarithmic graph of copy number versus the threshold cycle (Ct) for the dilution factor was plotted and used as a standard curve to determine the amount of viral DNA (copy number) in each μL of total DNA in a reaction mix.

The qPCR analyses were performed with the MX300P real-time thermocycler (Stratagene, La Jolla, CA) using Power SYBR Green PCR Master Mix (Applied Biosystems, Foster City, CA). The amplification reactions were performed in 50 μL containing 0.2 μM forward and reverse primers, ultrapure water and the optimum amount of DNA template as determined in titration experiments for the respective viruses (data not shown). Each virus was tested in a separate 96-well plate in which the first row contained the 10-fold serially diluted plasmid DNA for the standard curve.

### 2.4 DNA ploidy levels

To determine DNA ploidy levels of ‘Florida Lanai’ infected with different geminiviruses, leaf samples were taken from plants showing symptoms of TYLCV, ToMoV and BCTV as well as from mock-inoculated and healthy plants for comparison. Three biological replicas were collected for each treatment. Ploidy levels were determined using an Accuri™ C6 Flow Cytometer (BD Biosciences). Nuclei suspensions were prepared by chopping ca. 200 mg of fresh leaf tissue with a sharp razor blade in chopping buffer (3 mL Galbraith buffer + 10 μL β-ME and 2 μL RNase A) for 5 min on ice. Buffer preparation and other processes were done according to the BD Accuri™ C6 Flow Cytometer user manual. Data were plotted using internal BD Accuri C6 software, and peak positions and relative ploidy indices determined.

### 2.5 Statistical analysis

Statistical analysis was performed using Microsoft Excel (Office 2013). Analysis was performed using paired, two-tailed Student's *t*-test and p < 0.05 as the statistically significant cutoff. One-way analysis of variance was used to establish differences among group means and the Least significant difference (LSD) test was used in pairwise comparison to analyze differences between means.

## 3 Results

**Fig. 1:**
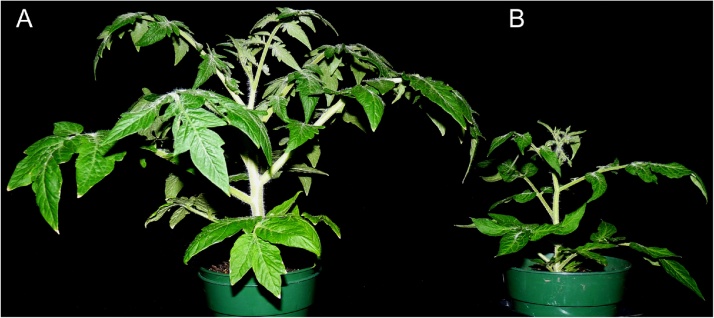
Comparison at 45 day-old **A:** Florida Lanai and **B:** Micro-Tom tomato varieties.

Three inoculation protocols were used: agroinoculation, particle bombardment and whitefly transmission, to inoculate ‘Florida Lanai’ plants with 5 diverse geminiviruses. A characterization of the effects of inoculation with each virus onto ‘Florida Lanai’ was performed. Also, seed transmission was determined for four of the viruses. A comparison between 'Florida Lanai' and Micro-Tom is shown for healthy plants (Fig. 1).

### 3.1 Symptom expression

**Fig. 2:**
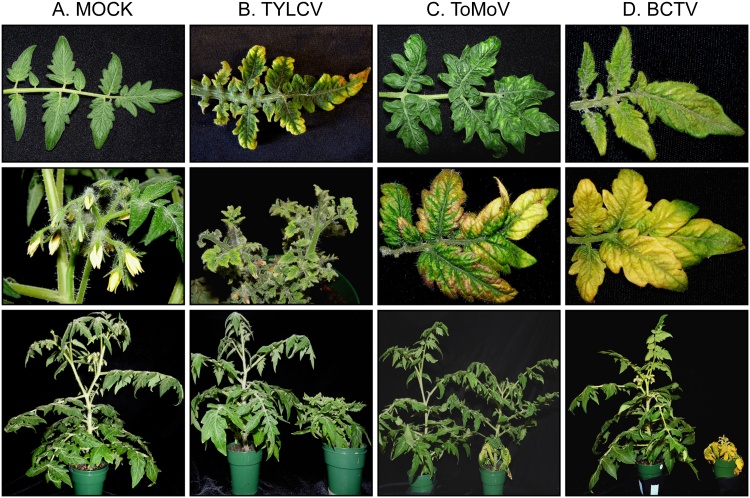
Symptoms observed on Florida Lanai plants mock- and agro-inoculated with TYLCV, ToMoV and BCTV.**A:** Mock-inoculated plant showing a healthy leaf, healthy flowers and a healthy plant (top to bottom). **B:** TYLCV inoculation showing chlorotic leaf margins, severe leaf size reduction, flower abscission and severe height reduction. **C:** ToMoV inoculation displaying bright yellow mottling in upper leaves, severe yellowing of lower leaves and medium plant height reduction. **D:** BCTV inoculation with general yellowing with mixed shades of green at early stages of infection, deep yellowing of the whole plant and very severe stunting at late stages of infection’.

Agroinoculation was a very efficient method for inoculating ‘Florida Lanai’ with TYLCV, ToMoV and BCTV resulting in 100% infection. Typical symptoms were observed in plants inoculated with these three viruses (Fig. 2A–D). Symptoms started to appear as early as 4 dpi for TYLCV and ToMoV and 7 dpi for BCTV. There were no observable symptoms in plants inoculated with TGMV or CaLCuV (data not shown) and no virus was detected by PCR. The failure of the TGMV to induce symptoms in tomato is well documented and we may have some issues with our agrobacterium inoculum (Wyant et al., 2012).

When young plants were agroinoculated with TYLCV (28 days after planting), the plants showed chlorotic leaf margins, upward leaf curling, severe leaf size reduction and flower abscission (Fig. 2B). When older plants were inoculated with TYLCV (45 days after planting), symptoms were limited to middle and upper leaves and ca. 85% of the floral buds were lost by abscission. Other symptoms included swelling of veins and severe stunting.

Plants agroinoculated with BCTV developed a general yellowing mixed with green at early stages of infection that progressed to deep yellow at advanced stages (Fig. 2D). Leaves were stunted, thicker and crisp with swollen veins. BCTV-infected plants generally exhibited severe stunting (Table 3 and Fig. 5). Approximately 25% of the plants infected at an early growth stage (28 days after planting) exhibited root decay and were dead by 45 dpi (data not shown), while the remaining plants did not recover or produce flowers. Plants infected later (45 days after planting) produced a few flowers, which did not open and dropped before fruit set.

**Table 3:** Comparison between infected and healthy plants for the change in height at different days after inoculation.

	Mean (cm)^a^	P-value^b^	% of height reduction
Mock			
7 dpi	2.65 ± 0.66		
14 dp1	5.27 ± 1.28		
21 dpi	7.68 ± 1.56		
28 dpi	8.58 ± 1.31		
35 dpi	11.1 ± 1.26		

TYLCV			
7 dpi	1.05 ± 0.38	≤0.001	60.4
14 dpi	2.09 ± 0.54	≤0.001	60.6
21 dpi	2.52 ± 0.60	≤0.001	67.2
28 dpi	2.99 ± 0.62	≤0.001	65.2
35 dpi	4.31 ± 0.65	≤0.001	61.0

ToMoV			
7 dpi	1.79 ± 0.63	0.008	32.5
14 dpi	3.94 ± 1.04	0.02	25.8
21 dpi	6.15 ± 1.64	0.05	19.9
28 dpi	8.00 ± 0.99	0.28	6.76
35 dpi	10.4 ± 1.42	0.27	6.15

BCTV			
7 dpi	1.93 ± 0.64	0.008	28.3
14 dp1	2.07 ± 0.72	0.002	62.1
21 dpi	2.16 ± 0.73	≤0.001	72.5
28 dpi	2.36 ± 0.87	≤0.001	72.8
35 dpi	2.88 ± 0.15	≤0.001	73.3

a Mean±S.D, n = 10.

b Significance level (P ≤ 0.05).

**Fig. 3:**
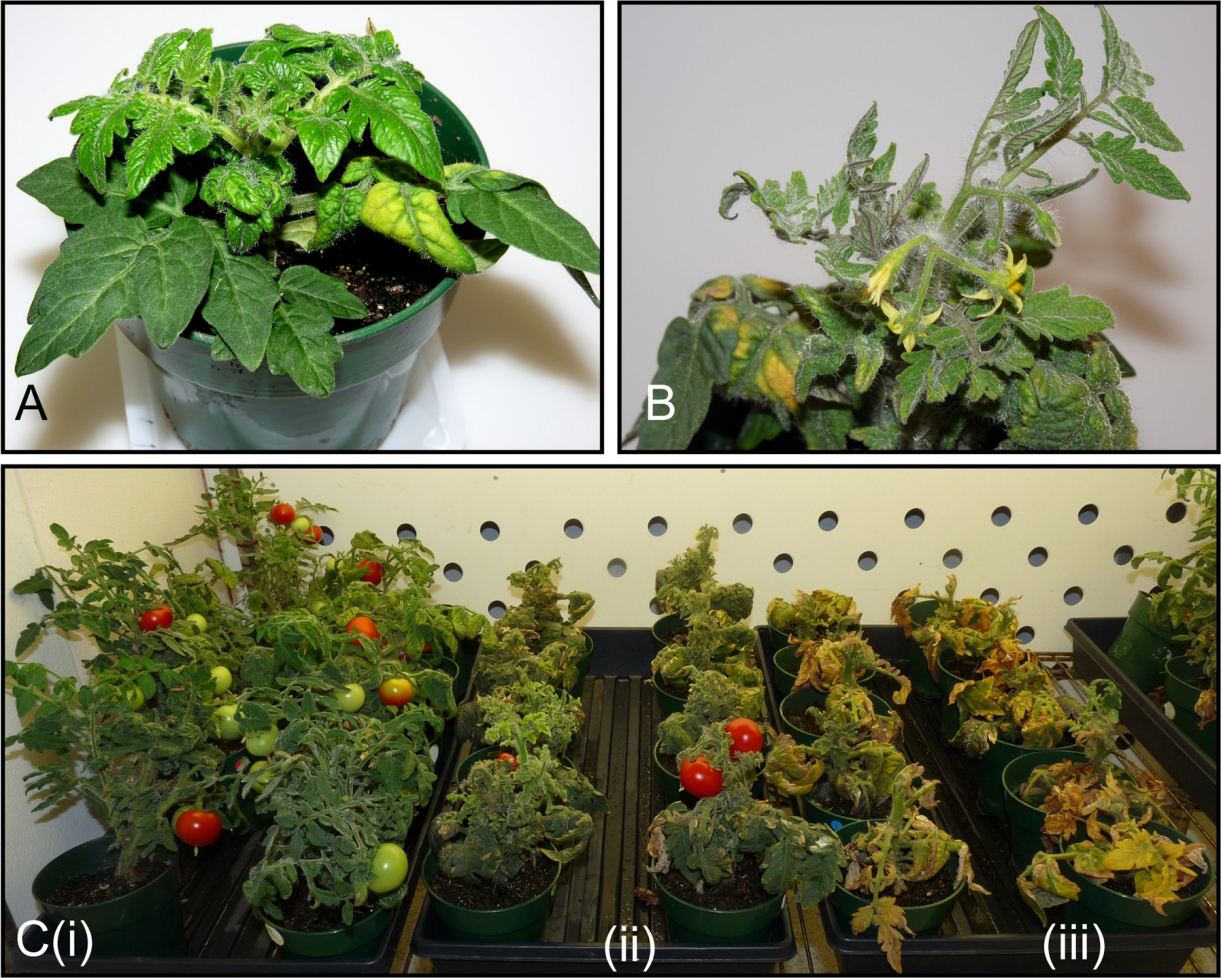
Florida Lanai recovering from ToMoV infection. **A:** Infection at 14 dpi, **B:** Infection at 28 dpi, **C:** Impact of recovery on yield (i) ToMoV, (ii) TYLCV and (iii) BCTV.

Plants agroinoculated with ToMoV typically developed a bright yellow chlorotic mottling on younger leaves and severe yellowing, leaf deformation and upward curling on lower leaves (Fig. 2C). Compared to plants infected with TYLCV or BCTV, ToMoV-infected plants showed only moderate stunting, less flower abscission and a smaller reduction in fruit (Figs. 2, 3, and Table 3). During ToMoV infection, the yellow chlorotic symptoms observed from 5 to 14 dpi changed to a recovery phenotype in which new leaf growth was symptomless and the plant grew faster producing many flowers and fruit (Fig. 3 and Table 3). ToMoV DNA was detected by PCR in leaves showing the recovery phenotype.

**Fig. 4:**
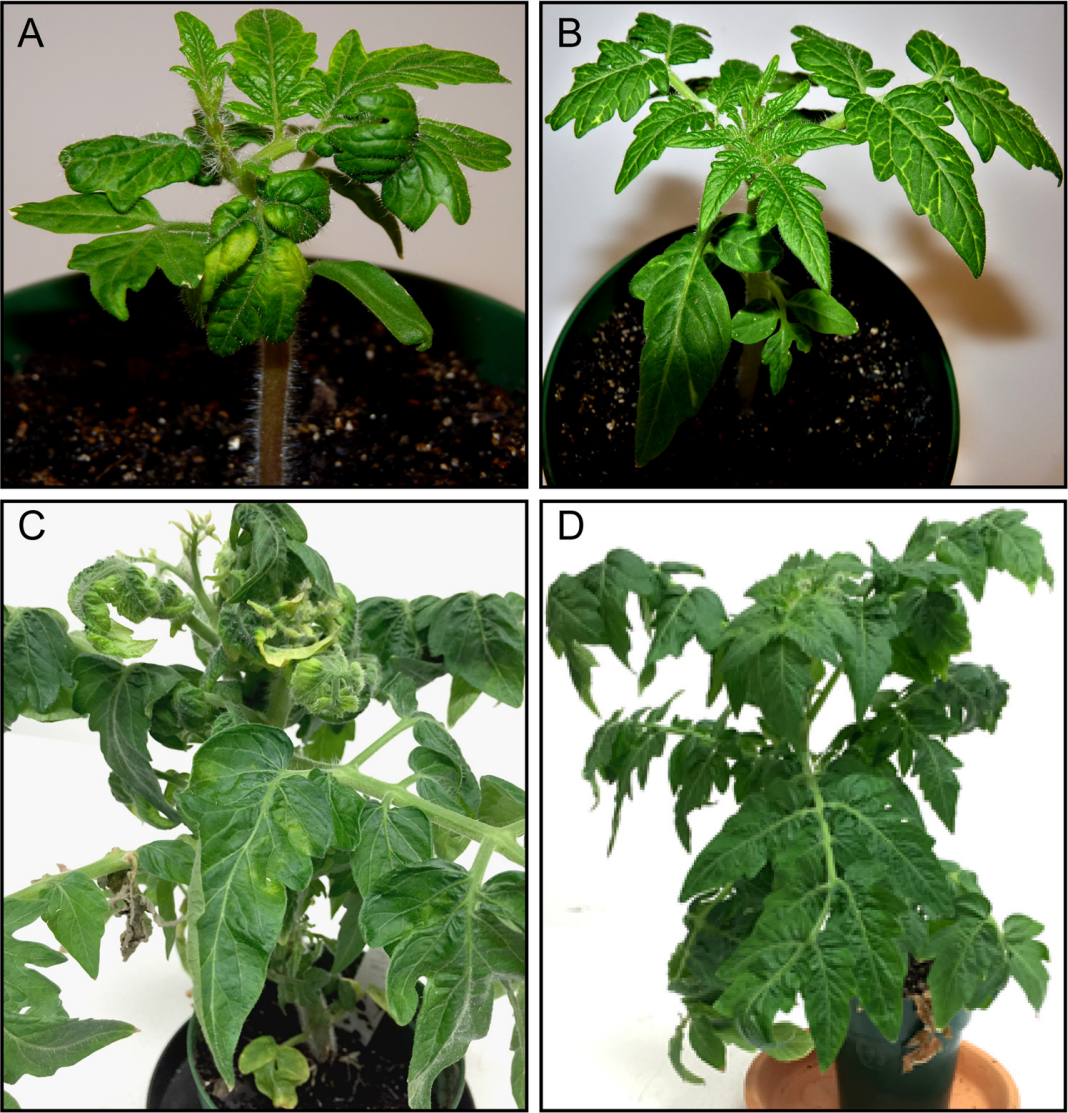
Symptoms on inoculated Florida Lanai by biolistics using **A:** ToMoV and **B:** TGMV. Bottom row: Florida Lanai infected by whitefly transmission using **C:** TYLCV and **D:** ToMoV, showing severe and very mild symptoms respectively.

**Fig. 5:**
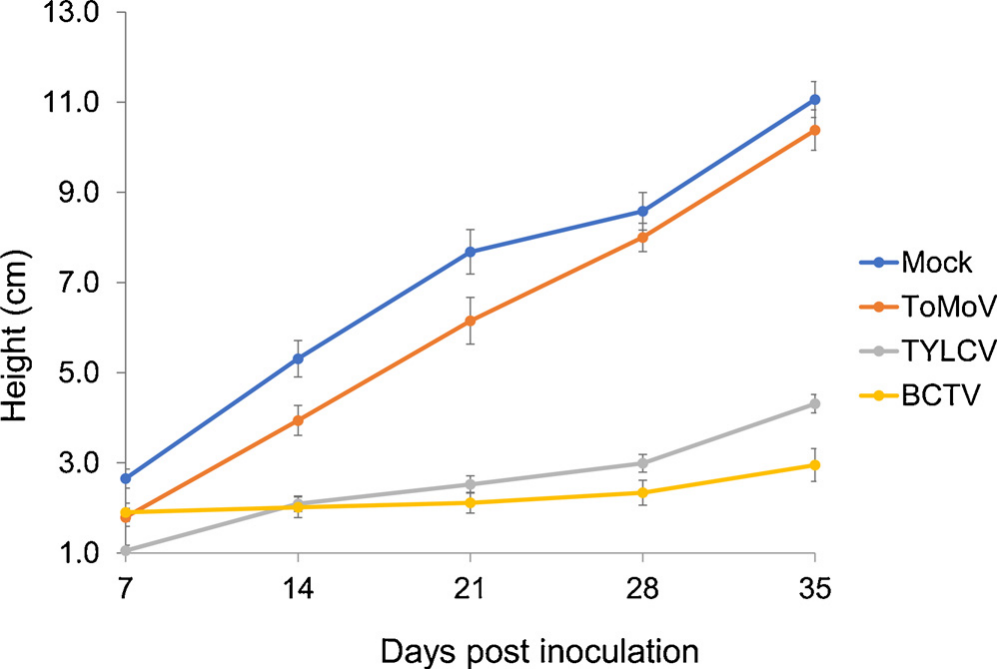
Change in plant height for Florida-Lanai tomato plants infected with TYLCV, ToMoV, BCTV and mock at different days post inoculation. Vertical bars represent the standard error (SE) of the means. N = 10 for all treatments.

Particle bombardment led to the infection of two of the five viruses used in this study. Virus symptoms were observed in 100% of the ‘Florida Lanai’ plants inoculated with ToMoV or TGMV by bombardment. No infected plants were observed in equivalent experiments using plasmids corresponding to TYLCV, BCTV or CaLCuV. Plants bombarded with ToMoV developed symptoms indistinguishable from those in agroinoculation experiments (Fig. 4A). Bombardment of TGMV DNA resulted in bright yellow coloration along veins (Fig. 4B). In comparison, TGMV inoculated plants (*N. benthamiana*) exhibited chlorotic mottling, leaf curling or spiral distortion, which was not observed in Florida Lanai (data not shown).

We tested TYLCV and ToMoV in whitefly transmission assays. Based on symptoms and PCR analysis, TYLCV was successively transmitted by viruliferous whiteflies from a TYLCV-infected source plant to a healthy Florida Lanai. By 30 days after introduction of viruliferous whiteflies, the target plants exhibited chlorotic leaf margins, upward curling of leaves, reduced leaf size and others symptoms characteristic of TYLCV infection described above (Fig. 4C). Whitefly transmission of ToMoV resulted in a very mild mottling on leaves (Fig. 4D).

A recent study (Kil et al., 2016) reported that geminiviruses can be transmitted through seed collected from TYLCV-infected plants. We produced seed from fruit collected from plants infected with TYLCV, ToMoV, BCTV or TGMV. After washing carefully with water, the seeds were planted and F1 and F2 progeny plants were examined for symptoms and viral DNA. None of the plants developed symptoms, and PCR assays did not detect viral DNA in any of the plants. These results showed that the geminiviruses we tested are not transmitted through ‘Florida Lanai’ seed.

### 3.2 Virus titer

Analyses of virus titer by conventional PCR or qPCR used total DNA extracted from leaves of ‘Florida Lanai’ plants. Primer pairs (Table 2) were optimized to amplify viral DNA at an annealing temperature of 58 °C. The TYLCV, ToMoV and BCTV standard curves for qPCR were linear in the range of 50 (1:10 dilution) to 5 × 10^−6^ ng (1:1 × 10^6^ dilution) per reaction (r^2^ > 0.99). We used 5 ng/reaction of total DNA for qPCR analysis of unknown viral DNA titers. This amount (5 ng) can be easily measured using a spectrophotometer.

Analysis of variance (ANOVA) showed that virus levels changed over time in all treatments (Fig. 7). There was a significant change up to 31 dpi in the means of viral load in plants infected with TYLCV (*F*_3,24_ = 5.30, *p* < 0.05), ToMoV (*F*_3,24_ = 7.28, *p* < 0.05) or BCTV (*F*_3,24_ = 3.08, *p* < 0.05) (Table 5A). Mean separation by a LSD test (Table 5B) showed that virus titer in plants infected with TYLCV increased significantly (α = 0.01) at 10, 17 and 24 dpi and then decreased at 31 dpi to a level similar to 10 dpi. Viral DNA increased in ToMoV-infected plants over a shorter window of time between 10 to 17 dpi (α = 0.05) and then declined (31 dpi, α = 0.01) consistent with the recovery phenotype. BCTV infected plants showed a continuous increase in virus titer from 10 to 31 dpi, with a significant increase at 31 dpi (α = 0.01). This correlates with observed continuous increase in symptom severity over time.

### 3.3 Plant height

Plants infected with TYLCV, ToMoV or BCTV were shorter than the mock-inoculated controls (Table 3 and Fig. 5). The reduction in height was highly significant (P < 0.05) for plants infected with BCTV or TYLCV at all sampling times. In contrast, ToMoV infection resulted in a significant height reduction during the initial stages of infection (7 and 14 dpi). During the later stages (21 and 28 dpi), ToMoV-infected plants underwent recovery and the heights of infected and mock-inoculated plants were not statistically different.

The establishment of BCTV infection was initially delayed (Table 3), but it ultimately caused the most severe disease symptoms. BCTV caused the largest reduction in the mean plant height (73.3%) at 35 dpi followed by TYLCV (67.2%) at 21 dpi. ToMoV had the smallest effect on Lanai growth. It recorded only 32.5% reduction in plant height at the initial stage of infection (7 dpi) before the plants recovered (Table 3).

### 3.4 Yield

Plants infected with TYLCV, BCTV or ToMoV showed reduced yields (Table 4). Reductions were most pronounced for TYLCV and BCTV, which were reduced for mean flower number, fruit number and fruit weight (g) per plant by 69.3, 93.5, and 95.3% respectively for TYLCV and 87.8, 100 and 100% respectively for BCTV. In contrast, ToMoV reduced the yield metrics by 8.5, 27.4 and 29.8%, respectively. The reductions were significant (P < 0.05) for numbers of flowers and fruit and fruit weight for plants infected with TYLCV and BCTV. The reductions in number of fruit and fruit weight were also significant for ToMoV-infected plants, but the reduction in number of flowers was not. From these results, it appears that TYLCV reduces the number of flowers and the proportion of flowers resulting in fruit due to excessive abscission, while ToMoV does not change the number of flowers produced by plants but increases flower abscission and causes a smaller reduction in fruit size. BCTV impairs the ability of plants to produce viable flowers and had a greater effect on yield than TYLCV. Plants infected early (21 days old) with BCTV produced very few flowers and none of them set fruit (Table 4). When older plants (at flowering, 45 days old) were infected with BCTV they formed flower buds that failed to open and eventually died (data not shown). Generally, all plants including the mock-inoculated controls produced many more flowers that set and produced fruit.

**Table 4:** Effect of TYLCV, ToMoV and BCTV on yield.

	Mean^a^	P-value^b^
Mock		
Mean flower number per plant	18.9 ± 4.15	
Mean fruit number per plant	6.20 ± 1.62	
Mean fruit weight per plant (g)	61.3 ± 14.4	

TYLCV		
Mean flower number per plant	5.80 ± 2.57	≤0.001
Mean fruit number per plant	0.40 ± 0.70	≤0.001
Mean fruit weight per plant (g)	2.91 ± 8.63	≤0.001

ToMoV		
Mean flower number per plant	17.3 ± 5.71	0.48
Mean fruit number per plant	4.50 ± 1.90	0.045
Mean fruit weight per plant (g)	43.0 ± 22.2	0.045

BCTV		
Mean flower number per plant	2.30 ± 0.2.21	≤0.001
Mean fruit number per plant	0 ± 0.00	≤0.001
Mean fruit weight per plant (g)	0 ± 0.00	≤0.001

a Mean±S.D, n = 10.

b Significance level (P ≤ 0.05).

**Table 5A:** One-way analysis of variance (ANOVA) for means of virus titer (copy number) for TYLCV, ToMoV and BCTV.

	Source of Variation	SS	df	MS	F	P-value	F crit
TYLCV	Between Groups	8.05E + 15	3	2.68E + 15	5.29	0.00602	3.01
	Within Groups	1.21E + 16	24	5.07E + 14			
	Total	2.02E + 16	27				

ToMoV	Between Groups	3.66E + 16	3	1.22E + 16	7.27	0.00123	3.01
	Within Groups	4.03E + 16	24	1.68E + 15			
	Total	7.69E + 16	27				

BCTV	Between Groups	2.11E + 14	3	7.03E + 13	3.08	0.04649	3.01
	Within Groups	5.48E + 14	24	2.28E + 13			
	Total	7.59E + 14	27

**Table 5B:** Difference between means and signiﬁcance of pairwise comparison (LSD) for means of virus copy number for TYLCV, ToMoV and BCTV at different days post inoculation. Differences indicated by * are significant at the α < 0.05 level and ** are signiﬁcant at the α < 0.01 level.

		10dpi	17dpi	24dpi	31dpi
TYLCV	10dpi	0	4.99E + 7**	2.42E + 7 ns	2.06E + 7 ns
	17dpi		0	4.75E + 8**	5.20E + 8**
	24dpi			0	4.48E + 7**
	31dpi				0

ToMoV	10dpi	0	5.66E + 7*	3.36E + 6 ns	4.52E + 7 ns
	17dpi		0	6.00E + 7*	1.02E + 8**
	24dpi			0	4.18E + 7 ns
	31dpi				0

BCTV	10dpi	0	2.94E + 6 ns	4.99E + 6 ns	7.48E + 6**
	17dpi		0	2.05E + 6 ns	4.54E + 6 ns
	24dpi			0	2.50E + 6 ns
	31dpi				00

dpi = days post inoculation.

ns = not significant.

### 3.5 DNA ploidy

**Fig. 6:**
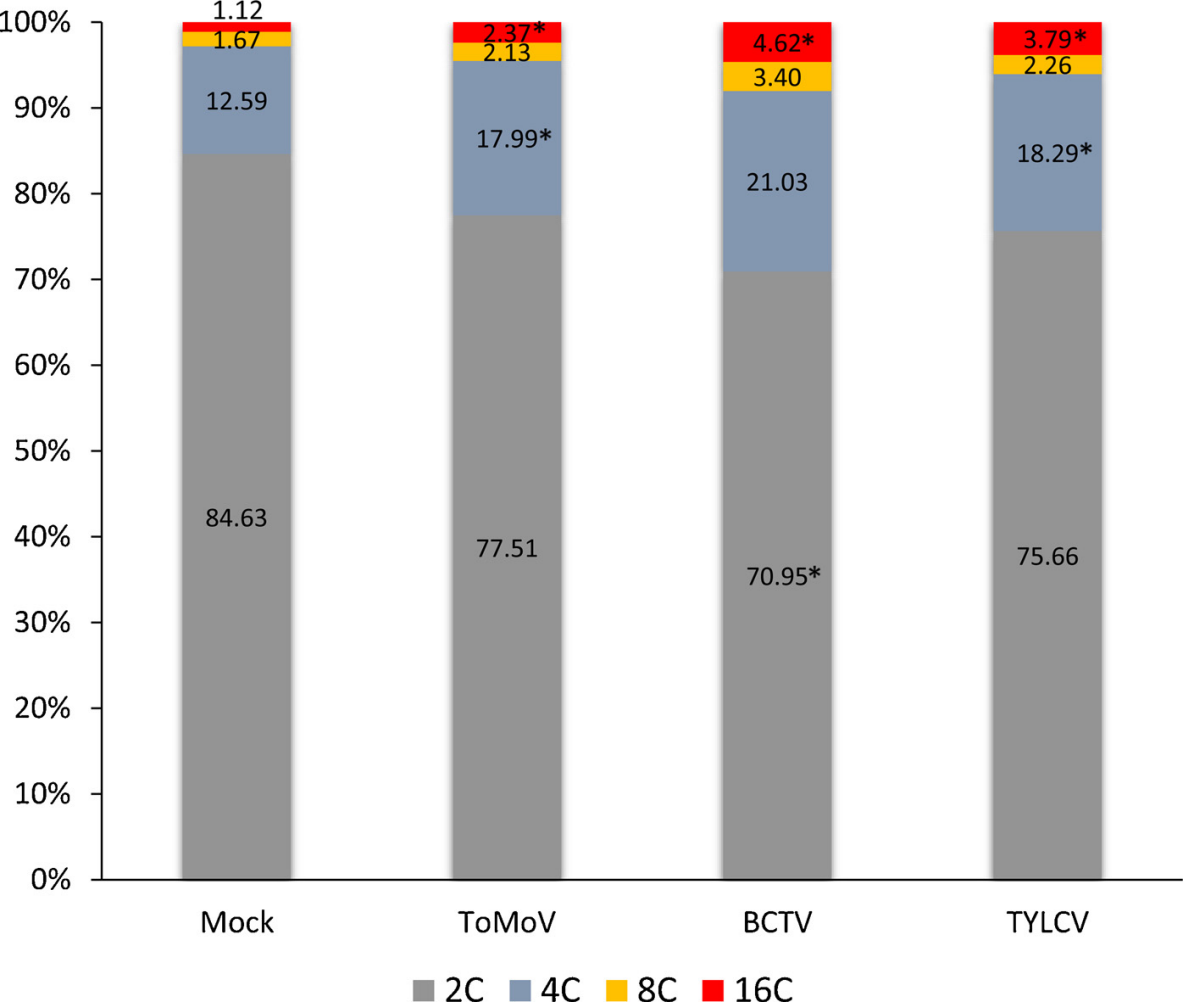
Histogram of the relative ﬂuorescence intensity of nuclei isolated from leaves of Lanai plants either mock-inoculated or inoculated with ToMoV, BCTV or TYLCV. The bars represent ploidy percentages for each treatment. Values indicated by * are statistically significant (P < 0.05).

Geminivirus infection modifies plant cell cycle controls to support replication of both viral DNA and plant chromosomes leading to increase genome ploidy (Ascencio-Ibáñez et al., 2008). Flow cytometry analysis of leaf nuclei of Lanai plants infected with TYLCV, ToMoV or BCTV and uninfected leaf controls showed four peaks corresponding to nuclei with 2C, 4C, 8C and 16C ploidy (Fig. 6). Virus infection changed the distribution of the peaks. A reduction in cells with lower ploidy (2C) and enrichment in cells with higher ploidy (4C, 8C and 16C) was observed during infection, with BCTV-infected plants displaying the largest changes in ploidy. The differences were found to be statistically significant for 4C and 16C for ToMoV and TYLCV infected plants, as well as for 16C for BCTV infected plants (Fig. 6).

**Fig. 7:**
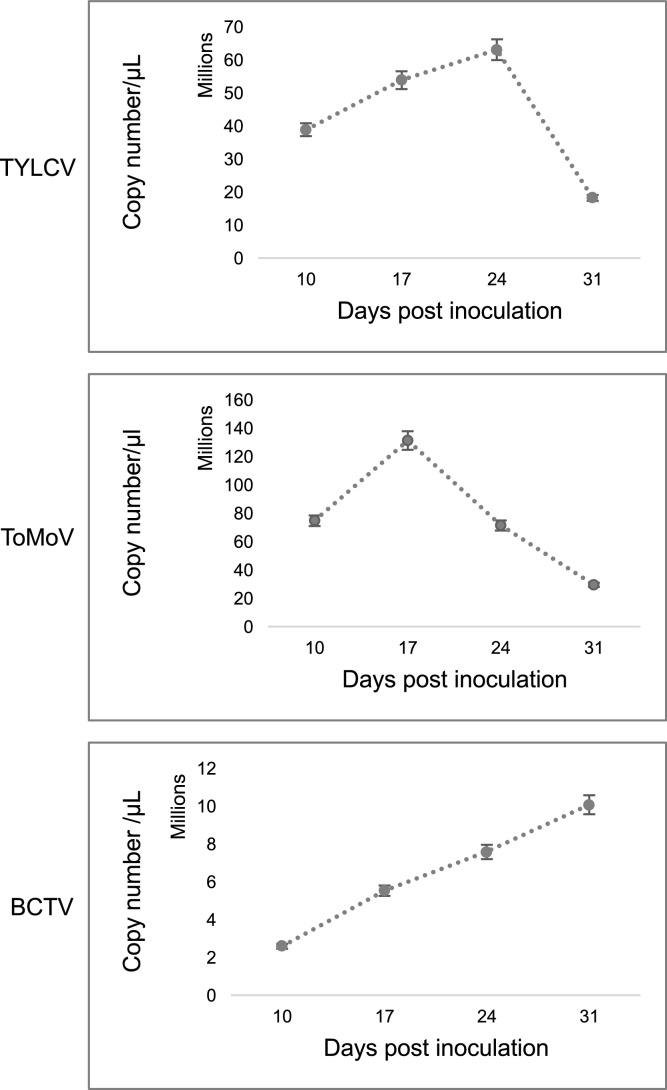
Changes in viral load over time for Florida Lanai infected with **A:** TYLVC, **B:** ToMoV and **C:** BCTV. Vertical bars represent the standard error (SE) of the means. N = 7 for all treatments.

## 4 Discussion

Other studies have highlighted the facility of virus transmission and ability to allow rapid replication as the most important characteristics of a good model system (Gergerich and Dolja, 2006; MacLean et al., 2011). TYLCV is an Old World monopartite begomovirus. ToMoV and TGMV are New World bipartite begomoviruses. Two of these viruses were identified and isolated from tomato, whereas TGMV was identified in tomato but propagated in and isolated from *N. benthamiana* (Cohen and Nitzany, 1966; Matyis et al., 1975; Costa, 1976; Buck and Coutts, 1985; Bisaro et al., 1982; Abouzid et al., 1992; Crespi et al., 1995). CaLCuV is a bipartite begomovirus in the Squash leaf curl clade from the New World, (Nawaz-ul-Rehman et al., 2009). CaLCuV was not cloned from or considered to be a pathogen of tomato. BCTV, which has a single-component genome, is a curtovirus with a broad host range that includes tomato (Bennett, 1971; Chen et al., 2010). ‘Florida Lanai’ plants were readily infected (100% success rate) by TYLCV, ToMoV and BCTV using agroinoculation, regardless of plant growth stage. ToMoV and TGMV were transmitted mechanically by a microdrop-sprayer, while ToMoV and TYLCV were transmitted by whiteflies (whitefly transmission of TGMV was not tested). Together, these results established that ‘Florida Lanai’ is a versatile model for studying geminivirus infection in tomato. The ability to infect the variety using more than one method of inoculation provides important alternatives when facilities or expertise to carry out other methods are lacking. The inability to inoculate TYLCV and BCTV by bombardment most likely reflects that they are largely phloem limited. With a few exceptions, phloem-limited viruses are not mechanically transmitted (Schneider, 1973; Esau, 1977, Rojas et al., 2001; Wyant, et al., 2012; Miozzi et al., 2014). Although recent reports showed geminivirus seed transmission (Kil et al., 2016), no seed transmission was detected for the viruses infecting Florida Lanai. Seeds were washed extensively prior to planting to minimize any potential contamination of the seed coat from surrounding fruit tissue, which may contain virus.

Another interesting observation is the ability of TGMV to infect Florida Lanai. Tomato is thought to be a non-host for TGMV even though the virus was originally found in tomato but maintained and cloned from *N. benthamiana* (Stenger et al., 1992; Matyis et al., 1975). Florida Lanai was readily infected with TGMV by biolistics with a 100% success rate. A previous study (Wyant et al., 2012) inoculated three tomato cultivars, including var. Moneymaker, with 25% efficiency.

The observation that ‘Florida Lanai’ plants displayed typical disease symptoms as early as 4 dpi, is an important characteristic of a good model plant. The short incubation period of the pathogen depends not only on the infectious agent but also on host susceptibility and ability to express symptoms (Dmitry and Van den Ackerveken, 2013). ‘Florida Lanai’ plants developed viral symptoms quickly, producing typical and distinct symptoms for different geminiviruses and enabling a systematic evaluation of the impact of different viruses in a common host. Walkey (1991) stated that good indicator plants respond to viral infections consistently and distinctively. These are important requirements for a model plant, especially when making a disease diagnosis, fulfilling Koch’s postulates or characterizing virus-host interactions. Quantifiable effects of virus infection on symptoms, leaf deformation, plant height, flower number, fruit number, fruit weight, effect on roots, and DNA ploidy were detected.

We used flow cytometry to examine the effect of virus infection on plant ploidy. TYLCV, ToMoV or BCTV infection increased the number of cells with higher ploidy levels (4C, 8C and 16C) and reduced the number of cells with lower ploidy levels (2C). These results confirm previous reports of increases in ploidy in mature leaves during geminivirus infection (Ascencio-Ibáñez et al., 2008). The earlier study detected ploidy changes in CaLCuV which is not confinced to the phloem. Thus, it was surprising to detect significant ploidy changes for BCTV and TYLCV, both of which have been reported to be phloem-limited in tomato (Schneider, 1973; Esau, 1977, Rojas et al., 2001; Miozzi et al., 2014), and it will be interesting to characterize further the interactions of these two viruses with ‘Florida Lanai’.

The patterns of virus accumulation in ‘Florida Lanai’ plants infected with TYLCV, ToMoV and BCTV provide more evidence of its suitability as a model system. The patterns related clearly with the severity of symptoms exhibited by the plants, fitting the general concept that higher virus titer leads to more plant damage (Ponz and Bruening, 1986). The kinetics of virus accumulation for TYLCV and BCTV followed general virus infection patterns (Rom et al., 1993) and corresponded well with the development and maintenance of severe symptoms throughout the time course of infection. In contrast, ToMoV plants showed significant rise in viral load early in infection followed by a rapid decrease. This decline was associated with the disappearance of symptoms. Reduced virus accumulation and a recovery phenotype are thought to be the consequence of host defenses overcoming the virus (Covey et al., 1997; Ratcliff et al., 1999; Zhou et al., 2008; Ma et al., 2015). One of the main factors in the success of infection is the ability of a given virus to suppress plant silencing pathways (Qu and Morris, 2005; Hanley-Bowdoin et al., 2013; Pumplin and Voinnet, 2013). Our results suggested that TYLCV and BCTV have stronger silencing suppressing activities than ToMoV. The use of a common plant host provides an excellent system for studying these differences because it eliminates any effects due to potential differences between host silencing factors across plant species and varieties (Nie and Molen, 2015).

Conclusions: The Florida Lanai tomato variety is an excellent model system for studying and comparing tomato infecting geminivirus-host interactions. Using this model system, researchers can obtain reliable results quickly even when space is limited. ‘Florida Lanai’ plants are readily infected by different viruses, delivered using different methods, to produce distinct measurable symptoms. More than 60 geminivirus species infect tomato (Inoue-Nagata et al., 2016), and we tested only four here. Hence, there is a need for further studies to determine if more geminiviruses can infect Florida Lanai. We recommend Florida Lanai as an excellent tomato variety for use as a model system for agroinoculation studies of TYLCV, ToMoV and BCTV, for mechanical bombardment of ToMoV and TGMV, and for whitefly transmission for TYLCV and ToMoV. Researchers may find it useful to use Florida Lanai in virus transmission studies, disease epidemiology studies and when investigating various physiological phenomena.

## Conflicts of interest

The authors have no conflict of interest to declare.
